# In memoriam: Francisc Schneider (1933‐2017)

**DOI:** 10.1111/jcmm.14173

**Published:** 2019-02-07

**Authors:** Virgil Păunescu, Mihaela Zavolan, Tudor Oprea

**Affiliations:** ^1^ OncoGen Research Center Timișoara România; ^2^ Biozentrum, University of Basel Basel Switzerland; ^3^ Department of Internal Medicine UNM School of Medicine Albuquerque New Mexico

**Keywords:** clinical physiology, functional explorations, molecular physiology

## Abstract

This is the obituary for Francisc Schneider, who introduced clinical physiology in Romania and mentored generations of scientists.

Professor Francisc Schneider, MD PhD, was born on 27 July 1933. He grew up under three different regimes^1^ in Timişoara, Romania. In good conscience,^2^ he was fascinated by the complexity of life and the afterlife. After he studied at the University of Medicine and Pharmacy in Timişoara (1951‐1957), he began his medical career as General Practitioner in the village of Certeze‐Negreşti Oaş. Decades later, its citizens awarded him the title of Honorary Citizen, in recognition of his dedication and professionalism. He was the first to introduce the practice of Clinical Physiology^3^ in Romania, and served as founding Chief of the Clinical Physiology and Clinical Investigations Laboratory at the Timiş County Hospital, Timişoara (1973‐1999). He was Full Professor and Department Head at two different Romanian universities (Timişoara until 1998, then Arad until 2016). He was elected member in several professional societies and academies, in Romania and elsewhere, served as Chair of the Romanian Society of Physiological Sciences (1998‐2008), was widely recognized for his scientific monographs and essay books and was a dedicated husband and father. Above all, he was an extraordinary mentor.

In the fog of a past long gone and so different from the present, a steady, insulating presence, an always measured response. The turmoil of the world around never seemed to stir the air in the old office by river Bega. Books, articles, digests of scientific journals continued to trickle there, keeping open a window through which some of us could catch the whiff of knowledge, scientific progress, intellectual pursuit.

Without prescribed paths, expectations, or justifications, we could simply pursue our dreams. Among Prof. Francisc Schneider's accomplishments, the one that I was privileged to bear testimony to was his generous mentorship.

Rest in peace, domnule professor. ‐M. Z.

He passionately pursued Big Science. He sought to understand respiratory physiology and bronchial asthma pathophysiology, the physiology of cognition and the pathology of neurodegenerative disorders, and studied the immune system. He was the founder of the Romanian school of Clinical Investigations and defined their role in the practice of medicine. He was the Editor‐in‐Chief of “Physiology (*Fiziologia*),” the only Romanian journal of physiology^4^ (1990‐2017). While Professor Schneider distinguished himself as a beacon of leadership rooted in tradition for the local Physiology community, he was an innovator and founding leader for the local immunology community, opening the path to world‐class accomplishments. He served at the helm of the Hygiene and Public Health Institute of Timişoara (1993‐1997). Above all, Professor Schneider had an eye for gifted young talent, whom he guided selflessly for decades.

Stern and demanding, passionate and with a keen sense of humour, Professor Schneider knew how to guide his young collaborators through action and inaction, through words and silence, but above all with patience and on‐point advice. Among his mentees, we recall Adrian Bot, MD PhD, today CSO at Kite Pharmaceuticals, Los Angeles, California; Flavius Martin, MD, vice president, oncology, Amgen, Thousand Oaks, California; Carmen Panaitescu, current President of the Romanian Society of Physiology, who keeps lit the torch of tradition for Timişoara's school of physiology; and the authors, Mihaela Zavolan, Virgil Păunescu and Tudor Oprea. All of us continue to pursue Big Science with the same passion that Professor Schneider instilled in us.

At the 2017 Romanian Society of Physiology Congress, Professor Schneider received an Honorary Award for his contributions as founding Editor‐in‐Chief of the “Physiology (*Fiziologia*)” journal. Anchored in the history of Judaism and the history of the Banat region, Professor Schneider was at home in the universe of ideas. From Aimé Michel's Metanoia^5^ to Arthur Guyton's Physiology,^6^ from philosophy and mysticism to the interpretation of electroencephalograms and molecular physiology, Francisc Schneider was burning into the flame of knowledge. In a country where Caragiale has already been surpassed by the theatre of the absurd, Francisc Schneider was, in the most genuine sense, “a school pedagogue of the new school” (“un pedagog de şcoală nouă” in Romanian). His enduring legacy are the mentees he guided through their first scientific steps (Figure 1).

**Figure 1:**
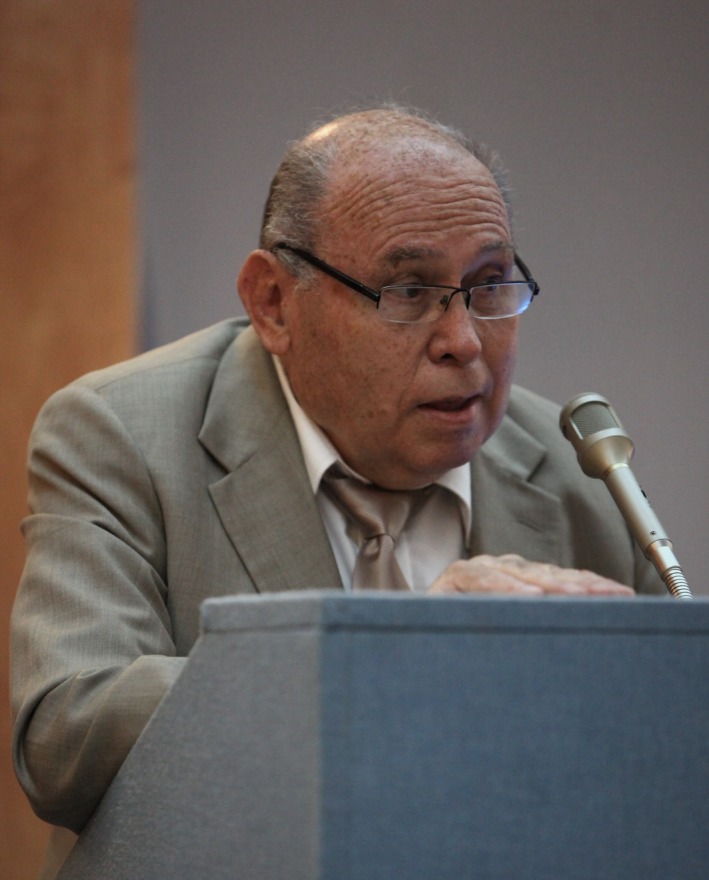
Professor Francisc Schneider, addressing former students at the Victor Babeș University of Medicine and Pharmacy, Timișoara, Romania, 18 June 2010

## CONFLICT OF INTEREST

The authors declare that they have no conflicts of interest.
